# Emergence of Rabies in Cape Fur Seals, South Africa

**DOI:** 10.3201/eid3210.260179

**Published:** 2026-10

**Authors:** Chuene Ernest Ngoepe, Lesley van Helden, Wonderful Shumba, Laura C. Roberts, Deon Kriel, Munangatire Mparamoto, Claude Taurai Sabeta

**Affiliations:** Agricultural Research Council–Onderstepoort Veterinary Research, Pretoria, South Africa (C.E. Ngoepe, M. Mparamoto, C.T. Sabeta); Western Cape Department of Agriculture, Elsenburg, South Africa (L. van Helden, L.C Roberts); Northern Cape Department of Agriculture, Environmental Affairs, Rural Development and Land Reform, Kimberley, South Africa (W. Shumba, D. Kriel); University of Pretoria, Pretoria (C.T. Sabeta)

**Keywords:** rabies, viruses, phylogenetic analysis, marine mammal, Cape fur seals, wildlife, South Africa

## Abstract

Rabies was diagnosed in Cape fur seals by using direct fluorescent antibody testing in South Africa, 2024. We investigated the origin of rabies virus in this species. Phylogenetic analysis revealed virus introduction from jackals into seals, followed by sustained viral transmission within the seal population and spillover into a domestic dog.

Rabies is a viral encephalitis caused by members of the *Lyssavirus* genus within the Rhabdoviridae family (*1*). Rabies virus (RABV) is a prototype species of the *Lyssavirus* genus and has a wide host range, including various Chiroptera and terrestrial Carnivora species (*2*). In southern Africa, RABV is maintained by domestic dogs and wildlife species such as black-backed jackals (*Lupulella mesomelas*), bat-eared foxes (*Otocyon megalotis*), aardwolves (*Proteles cristatus*), and yellow mongooses (*Cynictis penicillata*) (*3*).

In 2021, reports of unusually aggressive Cape fur seals (*Arctocephalus pusillus pusillus*) surfaced in South Africa; the behavior was attributed to domoic acid toxicity (*4*,*5*). On May 20, 2024, an unvaccinated domestic dog, henceforth referred to by its sample reference number, 170/24WC, from a walled suburban home in Cape Town was diagnosed with rabies using direct fluorescent antibody test (dFAT) on a brain tissue sample at the Agricultural Research Council–Onderstepoort Veterinary Research (Pretoria, South Africa). The owner suspected that a Cape fur seal had bitten the dog because the dog had a habit of approaching seals and sustained a bite wound while out of sight during an off-leash beach walk with the owner 2 weeks before the onset of clinical signs.

As part of the ensuing investigation, stakeholders in Cape Town were contacted to determine if any seals showing aggression or abnormal behavior had been observed or removed from beaches in the preceding month. A report stated that a Cape fur seal had been in a fight with dogs on Big Bay Beach in Cape Town on May 22, 2024, and suffered several injuries. The seal was captured by authorities and died in transit to a veterinary facility. A brain sample from this seal (sample reference 186/24WC) was obtained and tested positive for rabies by dFAT.

The Cape fur seal is the only resident pinniped species in southern Africa; an estimated 2 million animals are distributed from Baía dos Tigres in Angola to Algoa Bay, South Africa. The seals breed in colonies on land, where they frequently encounter people and face predation from terrestrial hunters such as the brown hyena (*Hyaena brunnea*) and the black-backed jackal (*6*). To our knowledge, the only previous confirmed case of rabies in a pinniped occurred in a ringed seal (*Pusa hispida*) in Svalbard, Norway in 1980 (*7*). We investigated to determine the origin and geographic spread of rabies virus infection in Cape fur seals in South Africa.

## The Study

Rabies is a notifiable disease in South Africa, and all suspected animal cases must be reported to government veterinary services. Beginning in June 2024, after RABV was confirmed in a Cape fur seal (186/24WC), all marine mammals showing neurologic signs were suspected to be rabid, and brain samples were collected from those that either died or could be captured and euthanized via lethal dose of pentobarbital or gunshot (Figure 1). In addition, opportunistic sampling of Cape fur seal or other marine mammal carcasses in remote areas of the coast was undertaken. We also sourced Cape fur seal brain samples collected during March 2023–May 2024 stored frozen and retrospectively tested for the RABV antigen, as previously described (*8*), and undertook RNA extractions, reverse transcription PCR, partial genome sequencing, and phylogenetic analyses of RABVs (Appendix).

**Figure 1:**
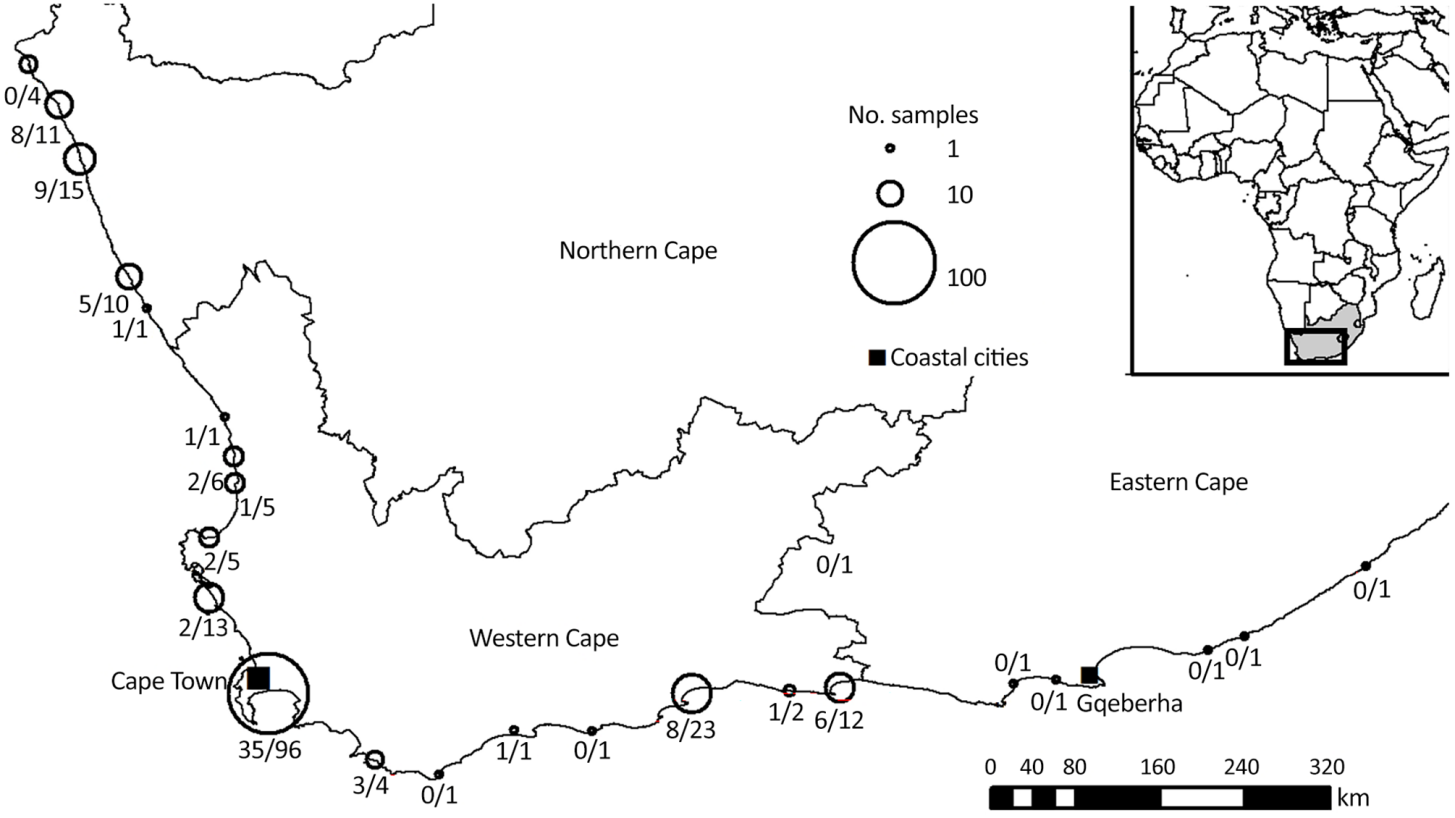
Sampling locations for study of emergence of rabies in Cape fur seals, South Africa. Numbers next to circles indicate no. positive/total no. samples. Inset map shows location of study area in Africa.

A total of 216 Cape fur seal brain tissue samples were collected along the South Africa coast during March 2023–November 2025, including 6 retrospective samples (Figure 1). Of those, 85 (39%) tested positive for lyssavirus antigen by dFAT; the remaining 131 (61%) tested negative. Positive samples were obtained from multiple locations along the coasts of the Northern and Western Cape provinces of South Africa. Of the 85 confirmed positive cases, 45 were found dead, and the other 40 were euthanized or shot. The earliest confirmed positive sample originated from Cape Town in September 2023 from a retrospective sample. The only other marine animal tested for rabies was an orca (*Orcinus orca*) found dead in St. Helena Bay in October 2024; results were negative.

A total of 51 RABVs detected in Cape fur seals and 1 from dog 170/24WC were available for sequencing. We deposited the generated nucleotide sequences in GenBank (Appendix Table). Phylogenetic analysis revealed 4 distinct clusters (A–D) of closely related viral sequences with posterior probability values of 1 (Figure 2). Cluster A comprised RABVs from 51 Cape fur seals and dog 170/24WC. The pairwise distance comparisons of RABVs in cluster A showed 99.79% nucleotide sequence identity. Furthermore, cluster A contained a subcluster (A1) with a posterior probability of 0.99. Cluster B consisted of RABVs from black-backed jackals and a bat-eared fox. Phylogenetic analysis showed that the RABVs in clusters A and B had a 98% nucleotide sequence identity and shared a common ancestor. Cluster C consisted of RABVs from bat-eared foxes and aardwolves, whereas cluster D contained RABVs from domestic dogs.

**Figure 2:**
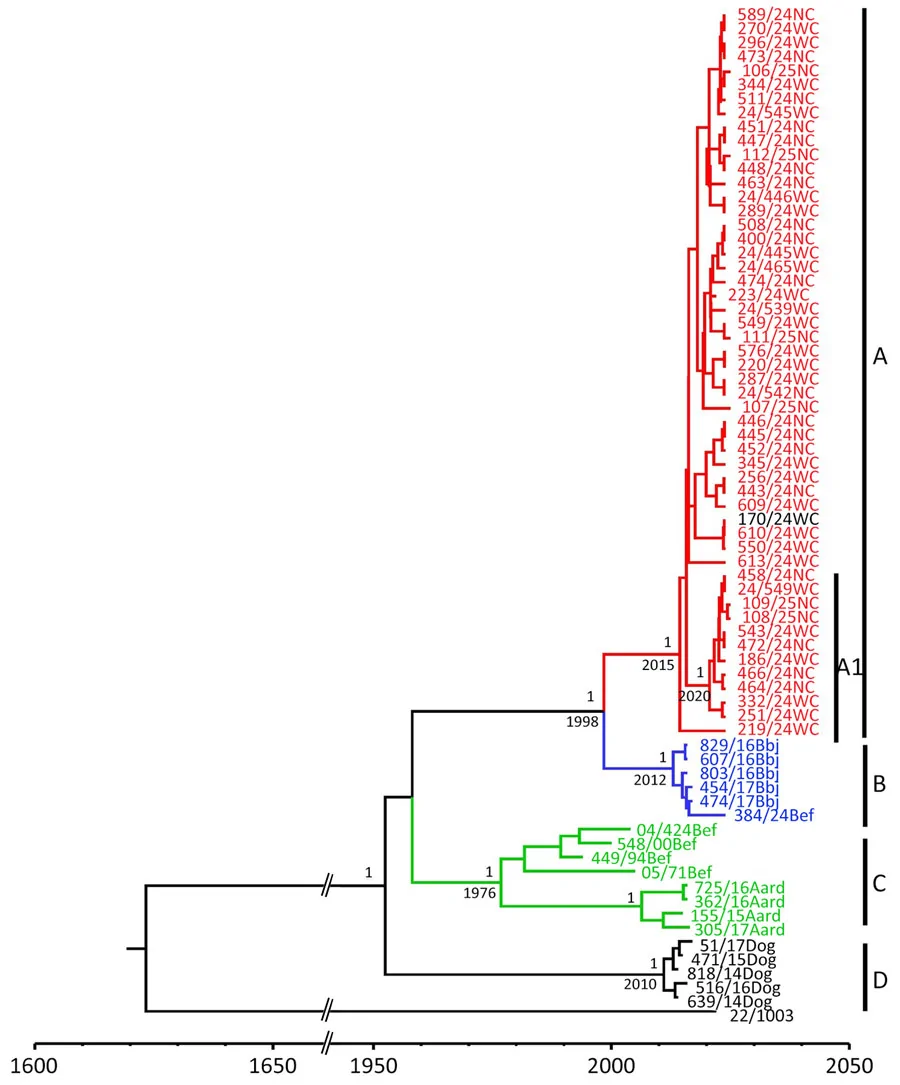
Timescale phylogenetic analysis for study of emergence of rabies in Cape fur seals, South Africa. Red font indicates sequences from Cape fur seals from this study, including retrospective samples; black text among seal samples indicates sample from a domestic dog (170/24WC) likely infected by contact with a seal. Green text indicates sequences from bat eared foxes (Bef) and aardwolves (Aard); blue text indicates sequences from black-backed jackals (Bbj) and one from bat eared fox; black text at bottom of tree indicates sequences from domestic dogs retrieved from GenBank.

BEAST evolutionary analysis (https://beast.community) showed that the mean rate of nucleotide substitutions for the RABVs included in the analysis was 3.7838 × 10^−4^ substitutions/site/year (95% highest posterior density 2.0322–5.6575 × 10^−4^ substitutions/site/year). The estimated time to the most recent common ancestor (tMRCA) of clusters A and B was 1998. The tMRCA of cluster A was estimated at 2015 and subcluster A1 at 2020 (Figure 2).

RABV typically spreads among animals of the same species, but direct interaction of a susceptible mammal with a reservoir host could result in cross-species transmission. In rare cases, such events have historically enabled RABV to evolve and adapt to new hosts (*3*), as observed in Cape fur seals in this study. All RABVs analyzed from seals had 99.79% sequence identity, indicating a single source of infection and subsequent viral transmissions within the seal population. RABVs introduced from multiple sources would show a lower degree of similarity (*3*,*9*). On the basis of available nucleotide sequence data, the black-backed jackal is the most likely source of introduction of RABV into the Cape fur seal population, given that tMRCA was closest for RABVs from the seals and the jackals. The RABVs in cluster A are estimated to have diverged from a common ancestor in 2015; thus, RABV was probably introduced into the seal population some years before it was laboratory confirmed. The large number of rabies-positive cases (n = 85) detected in this study that occurred during March 2023–November 2025 provides further evidence for a long period of seal-to-seal transmission, across almost the entire range of this species in South Africa. That spread was presumably aided by the ability of Cape fur seals to travel extensively in the ocean, resulting in a high degree of interaction across geographic areas (*10*). The RABV from dog 170/24WC clustered with the Cape fur seal RABVs, confirming the seal as its likely source of infection.

## Conclusions

Our findings demonstrate the ability of RABV to emerge in new host species and subsequently maintain transmission cycles in such hosts. However, this study is limited in its ability to estimate the prevalence of RABV within the Cape fur seal population because it relied on passive surveillance. Rabies virus remained undetected in Cape fur seal for years, highlighting the need for continuous surveillance in mammals displaying unusual behavior and neurologic signs to detect emerging lyssavirus variants. In addition, interventions that prevent cross-species transmission of RABV, such as large-scale parenteral vaccination of domestic dogs, should be continued and strengthened.

Appendix Additional information for study of emergence of rabies in Cape fur seals, South Africa.
