# Supplementary material for: Emergence of Rabies in Cape Fur Seals, South Africa
Source: Emerg Infect Dis. 2026 Oct;32(10):1686–9. doi: 10.3201/eid3210.260179 (PMC13632896; doi:10.3201/eid3210.260179)
Supplement: Supplementary file 1 [file 26-0179-Techapp-s1.pdf]

Article DOI: https://doi.org/10.3201/eid3210.260179

*EID cannot ensure accessibility for supplementary materials supplied by authors. Readers who have difficulty accessing supplementary content should contact the authors for assistance.*

# Emergence of Rabies in Cape Fur Seals, South Africa

## Appendix

### Viral RNA extraction, RT-PCR and phylogenetic analysis performed during the study

Total RNA was extracted from positive brain samples (n=51) using Tri-Reagent (Sigma Aldrich, USA) as per manufacturer's instructions. A reverse transcription polymerase chain reaction (RT-PCR) was performed by targeting the cytoplasmic domain of the glycoprotein gene and the G-L intergenic region of the viral genome as previously described (*1*). Since 1995, South African RABVs have been genetically characterized using Sanger sequencing of the G-L intergenic region of the viral genomes. Twenty additional South African rabies viruses (RABVs) nucleotide sequences available in the database were also included for phylogenetic analysis. Phylogenetic analysis was undertaken using a Bayesian Markov Chain Monte Carlo (MCMC) method in the BEAST X version 10.5.0 using a relaxed exponential clock and the sampling dates were included to investigate the linear evolutionary rates of RABVs (*2*). Runs were performed using the nucleotide substitution model general time reversible (GTR) with gamma plus invariable together with the constant size model as a coalescent tree prior. The phylogenetic analysis relied on three independent Markov chains sampled for 50 million states and a sampling frequency of 5000 was combined after discarding at least the first 10% burn. The posterior distributions were subsequently inspected using the Tracer software (version 1.7.1) to ensure adequate mixing and convergence before the associated statistics were summarized as a maximum clade credibility (MCC) tree using Tree Annotator version 10.5, and the final tree was displayed using FigTree version 1.4.4. The BEAGLE parallel computation library was used to enhance the speed of the likelihood calculations (*3*).

To investigate the linear evolutionary rates of RABVs in the selected panel of samples, the Bayesian Markov chain Monte Carlo in BEAST X version 10.5.0 was used (*2*) and incorporated information on the sampling date, year. For these analyses, runs were performed using the nucleotide substitution model general time reversible (GTR) with gamma plus invariable sites under strict molecular clock together with the constant size model as a coalescent tree prior. Fifty million chains were run (discarding the first 5 million steps), and trees were sampled every 5,000 steps. The BEAGLE parallel computation library was used to enhance the speed of the likelihood calculations (*3*). The resulting trees were summarized in an MCC tree, using Tree Annotator version 10.5 and the final tree was displayed in FigTree version 1.4.4.

## References


1. Sabeta CT, Mansfield KL, McElhinney LM, Fooks AR, Nel LH. Molecular epidemiology of rabies in bat-eared foxes (Otocyon megalotis) in South Africa. Virus Res. 2007;129:1–10. PubMed https://doi.org/10.1016/j.virusres.2007.04.024

2. Baele G, Ji X, Hassler GW, McCrone JT, Shao Y, Zhang Z, et al. BEAST X for Bayesian phylogenetic, phylogeographic and phylodynamic inference. Nat Methods. 2025;22:1653–6. PubMed https://doi.org/10.1038/s41592-025-02751-x

3. Ayres DL, Darling A, Zwickl DJ, Beerli P, Holder MT, Lewis PO, et al. BEAGLE: an application programming interface and high-performance computing library for statistical phylogenetics. Syst Biol. 2012;61:170–3. PubMed https://doi.org/10.1093/sysbio/syr100


**Appendix Table.** Rabies positive cases obtained from Cape fur seals in the Northern and Western Cape provinces of South Africa

| Item number | Laboratory number | Sampling date | Location | Province | Genbank Accession Number |
|---|---|---|---|---|---|
| 1 | 186/24 | 22-May-24 | Blouberg beach | Western Cape | PV165423 |
| 2 | 219/24 | 07-Jan-24 | Plettenberg Bay | Western Cape | PV165424 |
| 3 | 220/24 | 13-Jun-24 | Die Dam beach | Western Cape | PV165425 |
| 4 | 223/24 | 10-Aug-23 | Melkbosstrand | Western Cape | PV165426 |
| 5 | 251/24 | 08-Apr-24 | Plettenberg Bay | Western Cape | PV165427 |
| 6 | 256/24 | 26-Jun-24 | Muizenberg | Western Cape | PV165428 |
| 7 | 270/24 | 27-Jun-24 | Glencairn | Western Cape | PV165429 |
| 8 | 287/24 | 13-Jul-24 | Yzerfontein beach | Western Cape | PV165430 |
| 9 | 289/24 | 13-Jul-24 | Yzerfontein beach | Western Cape | PV165431 |
| 10 | 296/24 | 17-Jul-24 | Mossel Bay beach | Western Cape | PV165432 |
| 11 | 332/24 | 26-Jul-24 | Plettenberg Bay | Western Cape | PV165433 |
| 12 | 344/24 | 01-Aug-24 | Pearly Beach | Western Cape | PV165434 |
| 13 | 345/24 | 01-Aug-24 | Pearly Beach | Western Cape | PV165435 |
| 14 | 400/24 | 27-Aug-24 | Groen River Mouth | Northern Cape | PV165436 |
| 15 | 443/24 | 01-Sep-24 | Cliff point | Northern Cape | PV165437 |
| 16 | 445/24 | 01-Sep-24 | Cliff point | Northern Cape | PV165438 |
| 17 | 446/24 | 01-Sep-24 | Cliff point | Northern Cape | PV165439 |
| 18 | 447/24 | 01-Sep-24 | Cliff point | Northern Cape | PV165440 |
| 19 | 448/24 | 01-Sep-24 | Cliff point | Northern Cape | PV165441 |
| 20 | 451/24 | 01-Sep-24 | Cliff point | Northern Cape | PV165442 |

| Item number | Laboratory number | Sampling date | Location | Province | Genbank Accession Number |
|---|---|---|---|---|---|
| 21 | 452/24 | 01-Sep-24 | Cliff point | Northern Cape | PV165443 |
| 22 | 458/24 | 01-Sep-24 | Namakwa | Northern Cape | PV165444 |
| 23 | 463/24 | 01-Sep-24 | Kleinzee | Northern Cape | PV165445 |
| 24 | 464/24 | 01-Sep-24 | Kleinzee | Northern Cape | PV165446 |
| 25 | 466/24 | 01-Sep-24 | Kleinzee | Northern Cape | PV165447 |
| 26 | 472/24 | 01-Sep-24 | Kleinzee | Northern Cape | PV165448 |
| 27 | 473/24 | 01-Sep-24 | Kleinzee | Northern Cape | PV165449 |
| 28 | 474/24 | 01-Sep-24 | Kleinzee | Northern Cape | PV165450 |
| 29 | 24/539 | 19-Sep-24 | Velddrif | Western Cape | PV165451 |
| 30 | 24/542 | 18-Sep-24 | Sammy Beach | Northern Cape | PV165452 |
| 31 | 24/545 | 26-Jun-24 | Muizenberg | Western Cape | PV165453 |
| 32 | 24/549 | 17-Jul-24 | Mossel Bay beach | Western Cape | PV165454 |
| 33 | 543/24 | 23-Oct-24 | Clovelly | Western Cape | PV165455 |
| 34 | 549/24 | 03-Oct-24 | Muizenberg | Western Cape | PV165456 |
| 35 | 550/24 | 24-Oct-24 | Sonwabe beach | Western Cape | PV165457 |
| 36 | 511/24 | 15-Oct-24 | Glencairn | Western Cape | PV165458 |
| 37 | 508/24 | 10-Oct-24 | Lambert's Bay | Western Cape | PV165459 |
| 38 | 24/445 | 16-Aug-24 | V&A Waterfront | Western Cape | PV165460 |
| 39 | 24/446 | 12-Aug-24 | Hartenbos | Western Cape | PV165461 |
| 40 | 24/465 | 25-Aug-24 | Groot Brakrivier | Western Cape | PV165462 |
| 41 | 610/24 | 25-May-24 | Klein Brak beach | Western Cape | PV165464 |
| 42 | 589/24 | 25-Nov-24 | Port Nolloth | Northern Cape | PV165465 |
| 43 | 613/24 | 13-Nov-24 | Plettenberg Bay | Western Cape | PV165466 |
| 44 | 576/24 | 14-Nov-24 | Elands Bay | Western Cape | PV165467 |
| 45 | 609/24 | 10-Nov-24 | Buffelsbaai beach | Western Cape | PV165468 |
| 46 | 106/25 | 13 Mar 2025 | Namakwa | Northern Cape | PX718952 |
| 47 | 107/25 | 13 Mar 2025 | Namakwa | Northern Cape | PX718953 |
| 48 | 108/25 | 13 Mar 2025 | Namakwa | Northern Cape | PX718954 |
| 49 | 109/25 | 12 Mar 2025 | Kleinzee | Northern Cape | PX718955 |
| 50 | 111/25 | 12 Mar 2025 | Kleinzee | Northern Cape | PX718956 |
| 51 | 112/25 | 12 Mar 2025 | Kleinzee | Northern Cape | PX718957 |
